# Feeding characteristics and growth among children with prenatal exposure to Zika virus with and without microcephaly in the microcephaly epidemic research group pediatric cohort

**DOI:** 10.1186/s12887-024-04728-9

**Published:** 2024-04-29

**Authors:** Danielle Maria da Silva Oliveira, Demócrito de Barros Miranda-Filho, Ricardo Arraes de Alencar Ximenes, Ulisses Ramos Montarroyos, Elizabeth B. Brickley, Maria Helena Teixeira Pinto, Celina Maria Turchi Martelli, Regina Coeli Ramos, Thalia Velho Barreto de Araújo, Sophie Helena Eickmann, Paula Fabiana Sobral da Silva, Maria Durce Costa Gomes Carvalho, Olga Sophia de Sousa Martins, Ana Célia Oliveira dos Santos

**Affiliations:** 1 Speech Terapist and Epidemiologist Universidade de Pernambuco Santo Amaro- Recife- Pernambuco, Street Arnóbio Marques, Recife, 31050100-130 Brazil; 2 Universidade de Pernambuco, Recife, Pernambuco, Brazil; 3 Federal University of Pernambuco, Recife, Pernambuco, Brazil; 4 Instituto Aggeu Magalhães, Recife, Brazil; 5 London School of Hygiene & Tropical Medicine, London, UK; 6 Instituto Aggeu Magalhães, Recife, Pernambuco, Brazil

**Keywords:** Growth, Zika virus, Microcephaly, Feeding

## Abstract

**Objective:**

To describe the feeding characteristics and growth of children with prenatal exposure to Zika virus (ZIKV) from birth to 48 months.

**Design:**

Using data from the prospective Microcephaly Epidemic Research Group Pediatric Cohort (MERG-PC), children without microcephaly born to mothers with evidence of ZIKV infection during pregnancy (ZIKV-exposed children without microcephaly) and children with Zika-related microcephaly were compared using repeated cross-sectional analyses within the following age strata: birth; 1 to 12; 13 to 24; 25 to 36; and 37 to 48 months. The groups were compared in relation to prematurity, birth weight, breastfeeding, alternative feeding routes, dysphagia and anthropometric profiles based on the World Health Organization Anthro z-scores (weight-length/height, weight-age, length/height-age and BMI-age).

**Results:**

The first assessment included 248 children, 77 (31.05%) with microcephaly and 171 (68.95%) without microcephaly. The final assessment was performed on 86 children. Prematurity was 2.35 times higher and low birth weight was 3.49 times higher in children with microcephaly. The frequency of breastfeeding was high (> 80%) in both groups. On discharge from the maternity hospital, the frequency of children requiring alternative feeding route in both groups was less than 5%. After 12 months of age, children with microcephaly required alternative feeding route more often than children without microcephaly. In children with microcephaly, the z-score of all growth indicators was lower than in children without microcephaly.

**Conclusions:**

Children with Zika-related microcephaly were more frequently premature and low birth weight and remained with nutritional parameters, i.e., weight-for-age, weight-for-length/height and length/height-for-age below those of the children without microcephaly.

## Introduction

Children with congenital Zika virus (ZIKV) infection may present with severe brain damage, neuropsychomotor developmental delays, and dysphagia, that can contribute to compromised nutrition and growth over the early life course [1, 2]. In addition to their role in causing swallowing disorders, ZIKV-related neurological impairments may also lead to challenges with introducing solid foods and with acquiring motor skills for performing the eating-related tasks required to attain an appropriate diet. Some recent studies have suggested that the greater the severity of ZIKV-related microcephaly, the greater the impairment of neurodevelopment in children [3, 4].

The growth patterns of children with neurological impairment often differ from those of the typical pediatric population. Between 2001 and 2012, Araújo and Silva observed, in a cross-sectional study with 187 children with cerebral palsy not attributable to Zika, that the weight found was considered below the 50th percentile in most individuals, both on the curve for head circumference (HC) (56%) and on the CDC curve (86%) [5].

There are still few studies on the growth patterns of children with CZS. In general, these are studies with small samples, and with children evaluated up to a maximum of 2 years of age. Silva et al. reporting the early growth of 48 infants with probable CZS, most of them with microcephaly (86.7%), followed to age 1–8 months. They found a decreasing of –0.08 and –0.16 per month for the mean weight and length z-scores, respectively [6]. In another Brazilian study, França et al. found a significant difference in relation to height and weight measurements when compared 8 children with microcephaly with 16 children as a typical control group, both with an average age of 20.5 months [7]. Santos et al., in a case series with 65 infants with microcephaly, describe that 20% of them had a weight-age (W/A) deficit and 23% had a length/height-age (H/A) deficit at birth and that up to 12–23 months, 41.5% of them had a W/A deficit and 56.9% had H/A deficit [6].

 Using longitudinal data collected prospectively from the Microcephaly Epidemic Research Group Pediatric Cohort (MERG-PC) [8] this study aims to describe the feeding characteristics and growth of children without microcephaly born to mothers with evidence of ZIKV infection during pregnancy (ZIKV-exposed children without microcephaly), comparing with a group with Zik-related microcephaly, from birth to 48 months.

## Methods

As described in detail in the cohort profile paper [8], the MERG-PC follows up children with prenatal ZIKV who were born with and without apparent symptoms of Congenital Zika Syndrome (CZS) during the 2015–2016 ZIKV epidemic in Pernambuco, Brazil. The current study, which includes follow-up until March 2020, analyzes repeated cross-sections based on age groups of the children; hence, the number of children assessed varies across the specific age strata and therefore the children assessed at each moment were not necessarily the same.

The study sample included children without microcephaly born to mothers with evidence of ZIKV infection during pregnancy (ZIKV-exposed children without microcephaly) and children with Zik-related microcephaly. The group with microcephaly included children who (i) were born during the microcephaly epidemic between May 2015 and April 2017, (ii) were diagnosed with microcephaly at birth or during the follow-up period, and (iii) had laboratory evidence of ZIKV infection or phenotypic features and imaging abnormalities consistent with CZS. These criteria, take into account laboratory evidence of ZIKV infection and TORCHs and central nervous system imaging, were adapted from França et al [9]. The group without microcephaly included children who (i) were born between December 2015 to June 2017 to mothers with rash during pregnancy, (ii) were never diagnosed with microcephaly during follow-up, and (iii) had laboratory evidence of ZIKV infection during pregnancy.

Microcephaly was defined as a head circumference (HC) z-score ≤ -2 for sex and age. HC z-score calculations at birth were based on INTERGROWTH-21st curves; for children born preterm, a correction was made until the completion of 64 weeks of gestational age [10]. After birth for term children or after 64 weeks for preterm children, HC z-score calculations were based on World Health Organization (WHO) Anthro curves [11] Children’s HCs were evaluated at birth and during follow-up, and children who developed microcephaly postnatally were analyzed as part of the group of children with microcephaly.

Children’s exposure to ZIKV during pregnancy was based on a combination of longitudinal data from molecular (quantitative reverse transcription polymerase chain reaction [qRT-PCR]) and serological (immunoglobulin [Ig]M, IgG3, and plaque reduction neutralization [PRNT_50_]) testing for ZIKV in pregnant women and categorized as positive, suspected or negative. As previously described [12] the ZIKV-positive group was divided into three subcategories of robust, moderate and limited diagnostic evidence. Cases with robust evidence had a positive qRT-PCR test, seroconversion, at least two positive serologic tests in pregnancy, or one positive serologic test (i.e., IgM or IgG3) in pregnancy paired with a non-negative PRNT_50_ within six months post-pregnancy. Cases with moderate evidence had only one positive serologic test (i.e., IgM or IgG3) in pregnancy, an indication of seroconversion by PRNT_50_ during pregnancy, or an equivocal PRNT50 test result in pregnancy paired with a positive PRNT_50_ within three months post-pregnancy. Cases with limited evidence had a positive PRNT_50_ in pregnancy or within 6 months post-pregnancy or an indication of PRNT_50_ seroconversion during the 2 to 3 months post-pregnancy. Suspected cases had PRNT_50_ titers ≥ 20 and < 100 (in pregnancy or 1 month post-pregnancy) or a non-negative result (unspecified titer ≥ 20) during pregnancy or within 1 month post-pregnancy [12].

The children were assessed through multidisciplinary care cohort collective efforts, which took place monthly at the Centro de Reabilitação da Fundação Altino Ventura (FAV) and, whenever necessary, reassessed at the Hospital Universitário Oswaldo Cruz.

The groups were divided into the following age groups: birth, from 1 to 12 months, from 13 to 24 months, from 25 to 36 months and from 37 to 48 months.

The anthropometric profile of the children was established based on the Z-score for each variable, using the WHO growth curves [11] as a reference.

In the anthropometric assessment, weight, length and head circumference were measured as described below.

Children were weighed using an electronic baby-weighing scale with a maximum capacity of 15 kg and divisions of 5 g. When a child weighed more than 15 kg, a portable electronic scale was used, with a capacity of 180 kg.

Length was measured with the participation of two examiners (generally a mother and a professional), with an anthropometric ruler, with the child naked and barefoot, lying in dorsal decubitus on a flat surface. Due to the physical limitations of children with microcephaly, this technique was only used in children over 2 years of age, when the height would normally be measured while the child was standing.

The nutritional classification was performed using the WHO Anthro® [13] and the results were expressed in Z-scores, considering the length/height-for-age (H/A), weight- for-age (W/A), weight-for-height/length (W/H) and body mass index-for-age (BMI/A).

For the purpose of analysis, some categories of nutritional variables were grouped as described below:

- Weight-for-Age Z-score: Very low weight + low weight; appropriate weight + length/height.
- Length/Height-for-age: Very short + short.
- Weight-for-length/height: Marked thinness + thin; risk of overweight + overweight + obese.
- BMI-for-age: Marked thinness + thin; risk of overweight + overweight + obese.

Variables obtained at birth that could influence the nutritional status of the children were: prematurity, birth weight, breastfeeding on discharge from the maternity hospital and duration of breastfeeding. This information was collected at the first interview to avoid information bias. The groups of children were compared regarding the frequency of these variables.

Information on factors that may interfere with growth was obtained after birth until 48 months of age: food allergy; food intolerance; use of alternative feeding route; type of alternative feeding route, classified as nasogastric tube (NGT), nasoenteral tube (NET) or gastrostomy (GTT); and the presence and level of dysphagia. Dysphagia was defined and classified based on the PAD-PED [14] from a Clinical Speech-Language Pathology Assessment of Swallowing [14]. The two groups of children were compared regarding the factors described above in each cross-section.

### Ethics

The study was approved by the Research Ethics Committee and was conducted in accordance with the Declaration of Helsinki. Parents/guardians provided written informed consent for the children to participate.

### Outcomes

Feeding characteristics of study participants were evaluated based on data collected at birth/first evaluation and during the follow-up. Data were obtained using medical records, standardized data collection instruments administered to mothers by trained interviewers and evaluation by the research team. Variables obtained at birth that could influence the nutritional status of the children were: prematurity, birth weight, breastfeeding on discharge from the maternity hospital.

Additional information on factors that may influence growth was obtained after birth until 48 months of age and included data on: duration of breastfeeding, food allergies, food intolerances, use of and type of alternative feeding route (i.e., NGT, NET or GTT) and the presence and level of dysphagia. Dysphagia was defined and classified based on the PAD-PED [14] from a Clinical Speech-Language Pathology Assessment of Swallowing [15]. The person accompanying the child was weighed with the child on their lap and then alone, so that the weight of the child was obtained from the difference between the two weights. Length was measured with an anthropometric ruler and the participation of two examiners (generally a caregiver and a professional), with the child barefoot and lying in dorsal decubitus on a flat surface.

The nutritional classification was performed using the WHO Anthro® [13] and the results were expressed in z-scores, considering the length/height-for-age (H/A), weight- for-age (W/A), weight-for-height/length (W/H) and body mass index-for-age (BMI/A). For the purpose of analysis, some categories of nutritional variables were grouped as: i) weight-for-Age (z-score): very low weight + low weight, appropriate weight + length/height; ii) length/height-for-age: very short + short; iii) weight-for-length/height: marked thinness + thin, risk of overweight + overweight + obese; iv) BMI-for-age: marked thinness + thin, risk of overweight + overweight + obese.

### Statistical analysis

The statistical analysis was performed using STATA SE 14.2 (College Station, TX, USA). Categorical variables were summarized in absolute numbers and percentages. To compare the groups of children, the chi-square test was used. The Fisher's exact test was used when cells contained 5 or fewer observations. Continuous variables were summarized as means and standard deviation. To estimate the change in the HC (z-score), weight (z-score) and length/height (z-score over time) we used multilevel mixed effects linear regressions with child-specific random effects.

## Results

In the current study, data were analyzed in five cross-sections occurring at birth (*n* = 248), from 1 to 12 months (*n* = 99, median 9 months), from 13 to 24 months (*n* = 162, median 20 months), from 25 to 36 months (*n* = 175, median 30 months) and from 37 to 48 months (*n* = 8, median 39 months).

Among both children with and without microcephaly, the majority of children were born at term; however, prematurity was 2.4-times more frequent in the group with microcephaly (Table 1). The groups also differed in terms of birth weight, with the frequency of low birth weight being 3.4-times higher in children with microcephaly. No significant differences between children with and without microcephaly were observed regarding the use and period of breastfeeding and the way the children were fed when discharged from the maternity hospital. In assessments performed after 12 months, we observed that children with microcephaly used an alternative feeding route more frequently (approximately 18%) than children without microcephaly (approximately 1%) (Table 2); nevertheless, we note that one child without microcephaly was using NET at the time of the second assessment (i.e., during infancy). By the end of follow-up, four children with microcephaly were using a definitive alternative feeding route (i.e., GTT). No specific pattern of change in the BMI z-score was observed after the introduction of the alternative feeding route in these children. The frequency of reporting food allergy and food intolerance was low in both groups throughout the follow-up period (Table 2).

**Table 1:** Characteristics at birth and feeding type at birth and after discharge of children exposed to the Zika virus, with and without microcephaly, Pernambuco/Brazil, 2016–2020

Variables	Microcephaly		Without Microcephaly	
	N	%	N	%
**Prematurity (*n* = 211)**				
**No**	50	79.4%	135	91.2%
**Yes**	13	20.6%	13	8.8%
***P*-value**	*p*-value 0.001			
**Birth weight (*n* = 248)**				
**< 2500 g**	22	31.4%	16	9.0%
**> 2500 g**	48	68.6%	162	91.0%
***P*-value**	*p*-value 0.000			
**Breastfeeding (*n* = 243)**				
**Yes**	60	87.0%	161	92.5%
**No**	9	13.0	13	7.5%
***P*-value**	*p*-value 0.172			
**Duration of breastfeeding**				
≤**6 months**	40	58.0%	90	51.1%
**> 6 months**	29	42.0%	86	48.9%
***P*-value**	*p*-value 0.335			
**Feeding Route at discharge from maternity hospital**				
***Mother’s breast (n* = *245)***				
**Yes**	37	54.4%	109	62.6%
**No**	31	45.6%	65	37.4%
***p-value* = *0.239***				
***Bottle (n* = *242)***				
**Yes**	4	5.9%	3	1.7%
**No**	64	94.1%	171	98.3%
***p-value* = *0.083***				
***Nasogastric tube (n* = *242)***				
**Yes**	2	2.9%	3	1.7%
**No**	66	97.1%	171	98.3%
***p-value* = *0.109***				
***Nasoenteral tube (n* = *242)***				
**Yes**	1	1.5%	0	-
**No**	67	98.5%	174	100%
***Beaker***				
**Yes**	6	8.8%	10	5.8%
**No**	62	91.2%	164	94.3%

**Table 2:** Factors associated with the outcome that may influence the growth of children exposed to Zika virus, with and without microcephaly, Pernambuco/Brazil, 2016–2020

	**1 to 12 months**		**13 to 24 months**		**25 to 36 months**		**37 to 48 months**	
	**Microcephaly**		**Microcephaly**		**Microcephaly**		**Microcephaly**	
	**Yes**	**No**	**Yes**	**No**	**Yes**	**No**	**Yes**	**No**
**Food allergy**	*N* = 29		*N* = 115		*N* = 263		*N* = 93	
	*N* = 8 (27.6%)	*N* = 21 (72.4%)	*N* = 4 (3.5%)	*N* = 111 (96.5%)	*N* = 44 (17.11%)	*N* = 219 (82.89%)	*N* = 32 (34.4%)	*N* = 61 (65.6%)
** No**	7 (87.50%)	21 (100%)	3 (70.0%)	106 (95.5%)	43 (97.7%)	130 (93.5%)	29 (90.6%)	59 (96.7%)
** Yes**	1 (12.5%)	-----	1 (3.0%)	5 (4.5%)	1 (2.3%)	89 (6.5%)	3 (9.4%)	2 (3.3%)
***P*-value**	0.276 (Fisher)		1.000 (Fisher)		0.455 (Fisher)		0.335 (Fisher)	
**Food intolerance**	*N* = 29		*N* = 143		*N* = 181		*N* = 94	
	*N* = 8 (27.6%)	*N* = 21 (72.4%)	*N* = 33 (23%)	*N* = 110 (77%)	*N* = 44 (24.3%)	*N* = 137 (75.7%)	*N* = 33 (35.15%)	*N* = 61 (64.9%)
** No**	7 (87.5%)	20 (95.2%)	30 (90.9%)	105 (95.5%)	42 (95.5%)	129 (94.2%)	29 (87.9%)	59 (96.7%)
** Yes**	1 (12.5%)	1 (4.8%)	3 (9.1%)	5 (4.6%)	2 (4.6%)	8 (5.8%)	4 (12.1%)	2 (3.3%)
***P*-value**	0.483 (Fisher)		0.386 (Fisher)		1.000 (Fisher)		0.179 (Fisher)	
**Alternative feeding route**	*N* = 31		*N* = 145		*N* = 179		*N* = 94	
	*N* = 8 (25.8%)	*N* = 23 (74.2%)	*N* = 32 (22.1%)	*N* = 113 (77.9%)	*N* = 43 (24%)	*N* = 136 (76%)	*N* = 34 (36.2%)	*N* = 60 (63.8%)
** No**	8 (100%)	23 (100%)	27 (81.8%)	112 (99.1%)	36 (83.7%)	134 (98.5%)	28 (82.4%)	60 (100%)
** Yes**	------	------	5 (18.2%)	1 (0.9%)	7 (16.3%)	2 (1.5%)	6 (17.7%)	-----
***P*- value**	------		0.001 (Fisher)		0.001 (Fisher)		0.002 (Fisher)	
Type of alternative feeding route^*^	*N* = 31		*N* = 6		*N* = 8		*N* = 6	
			*N* = 5 (83.3%)	*N* = 1 (16.7%)	*N* = 6 (75%)	*N* = 2 (25%)	*N* = 6 (100%)	
** NGT**	----	----	---	----	----	2 (100%)	1 (16.7%)	----
** NET**	-----	-----	5 (100%)	1 (100%)	3 (50%)	----	1 (16.7%)	-----
** GTT**	-----	-----	----	----	3 (50%)	----	4 (66.7%)	-----
***P*-value**	-----		-----		0.036 (Fisher)		--------	
**Dysphagia**	*N* = 31		*N* = 143		*N* = 182		*N* = 94	
	*N* = 8 (25.8%)	*N* = 23 (74.2%)	*N* = 30 (21%)	*N* = 113 (79%)	*N* = 45 (24.7%)	*N* = 137 (75.3%)	*N* = 33 (35.1%)	*N* = 61 (64.9%)
** No**	4 (50%)	21 (91.3%)	13 (40.6%)	109 (96.5%)	17 (37.8%)	136 (99.3%)	11 (33.3)	61 (100%)
** Yes**	4 (50%)^*^	2 (8.7%)	17 (59.4%)	4 (3.5%)	28 (62.2%)	1 (0.7%)	22 (66.7%)	-----
***P*-value**	0.026 (Fisher)		< 0.001 (Fisher)		< 0.001 (Fisher)		< 0.001 (Fisher)	
**Level of dysphagia**	*N* = 31		*N* = 145		*N* = 182		*N* = 94	
	*N* = 8 (25.8%)	*N* = 23 (74.2%)	*N* = 30 (21%)	*N* = 113 (79%)	*N* = 45 (24.7%)	*N* = 137 (75.3%)	*N* = 33 (35.1%)	*N* = 61 (64.9%)
** Absent**	4 (50%)	21 (91.3%)	13 (40.6%)	109 (96.5%)	17 (37.8%)	136 (99.3%)	11 (33.3%)	61 (100%)
** Light**	3 (37.5%)	2 (8.7%)	2 (6.3%)	3 (2.7%)	6 (13.3%)	----	11 (33.3%)	----
** Moderate**	1 (12.5%)	---	10 (37.5%)	1 (0.9%)	13 (28.9%)	1 (0.7%)	7 (21.2%)	----
** Severe**	----	---	5 (15.6%)	----	9 (20.0%)	----	4 (12.1%)	-----
***P*-value**	0.026 (Fisher)		< 0.001 (Fisher)		< 0.001 (Fisher)		< 0.001 (Fisher)	

^∗^The children in this sample were aged between 5 months (1 child), 7 months (2 children), 11 months (1 child). NGT- nasogastric tube/ NET- nasoenteral tube/ GTT- gastrostomy

Among infants aged 1 to 12 months, the frequency of dysphagia was 50.0% in children with microcephaly and 8.7% in children without microcephaly. In the last cross-section, performed when the children were 37 to 48 weeks of age, the frequency of dysphagia was 66.7% in the group with microcephaly, while in the group without microcephaly there were no children with dysphagia (Table 2). The frequency of severe dysphagia in children with microcephaly increased from zero at birth to 15.6% in the second cross-section. In the final two cross-sections, the frequency of dysphagia was 20.0% and 12.1%, respectively (Table 2).

At birth, the frequency of underweight (i.e., very low weight and low weight) children was more than four times higher in the group with microcephaly, when compared to the group of children without microcephaly. This difference between the groups remained apparent throughout the follow-up period (Table 3). Regarding length/height-for-age, most children in both groups presented appropriate parameters, although we observed a higher percentage of children who were categorized as very short and short in the group with microcephaly in all assessments (Table 3).

**Table 3:** Anthropometric assessment of children exposed to Zika virus, with and without microcephaly, Pernambuco/Brazil, 2016–2020

	**At birth**		**1 to 12 months**		**13 to 24 months**		**25 to 36 months**		**37 to 48 months**	
	**Microcephaly**	**Microcephaly**			**Microcephaly**		**Microcephaly**		**Microcephaly**	
	**Yes** **(*n* = 70)**	**No** **(*n* = 178)**	**Yes** **(*n* = 40)**	**No** **(*n* = 59)**	**Yes** **(*n* = 37)**	**No** **(*n* = 125)**	**Yes** **(*n* = 44)**	**No** **(*n* = 131)**	**Yes** **(*n* = 33)**	**No** **(*n* = 53)**
**Weight-for-Age (z-score)**										
** Very low weight + low weight**	30 (43.5)	18 (10.3%)	20 (57.14%)	9 (16.36%)	11 (31.4%)	15 (12.6%)	18 (41.9%)	14 (11.1%)	15 (48.4%)	6 (11.3%)
** Appropriate weight + increased weight**	39 (56.5%)	156 (89.7%)	15 (42.9%)	46 (83.6%)	24 (68.6%)	104 (87.4%)	25 (58.1%)	112 (89.0%)	16 (51.6%)	47 (88.7%)
***p*-value**	< 0.001		< 0.001		0.009		< 0.001		< 0.001	
**Length/Height-for-Age**^a^										
** Very short + short**	18 (26.1%)	5 (2.9%)	13 (37.1%)	6 (10.9%)	4 (11.4%)	4 (3.4%)	6 (14.0%)	8 (6.4%)	9 (29.0%)	3 (5.7%)
** Appropriate**	39 (56.5%)	156 (89.7%)	15 (42.9%)	46 (83.6%)	24 (68.6%)	104 (87.4%)	25 (58.1%)	112 (88.9%)	16 (51.6%)	47 (88.7%)
***p*-valuer**	< 0.001		< 0.001		0.027		< 0.001		< 0.001	
**Weight-for-Length/Height**^a^										
** Marked thinness + Thin**	5 (10.6%)	10 (5.9%)	1 (2.9%)	4 (7.3%)	6 (17.1%)	5 (4.1%)	9 (21.4%)	8(6.3%)	2 (6.5%)	2 (3.8%)
** Eutrophic**	31 (66.0%)	113 (66.5%)	23 (65.7%)	30 (54.6%)	27 (77.1%)	80 (66.7%)	27 (64.3%)	77 (60.6%)	25 (80.7%)	25 (80.7%)
** Risk of overweight + overweight + obese**	11 (23.5%)	47 (27.7%)	11 (31.4%)	21 (38.2%)	2 (5.7%)	35 (29.2%)	6 (14.3%)	42 (33.1%)	4 (12.9%)	4 (12.9%)
***p*-value**	0.483		0.522 (Fisher)		0.001 (Fisher)		0.004		0.189 (Fisher)	
**Body Mass indexx-for-Age**^a^										
** Marked thinness + Thin**	14 (20.3%)	9 (5.6%)	3 (8.6%)	5 (9.1%)	3 (8.6%)	3 (2.5%)	6 (14.0%)	7 (5.6%)	2 (6.45%)	3 (5.7%)
** Eutrophic**	48 (69.6%)	127 (73.0%)	26 (74.3%)	31 (56.4%)	30 (85.7%)	81 (68.1%)	29 (67.4%)	76 (60.3%)	26 (83.9%)	34 (64.6%)
** Risk of overweight + overweight + obese**	7 (10.1%)	38 (21.8%)	6 (17.1%)	19 (34.6%)	2 (5.7%)	35 (29.4%)	8 (18.6%)	43 (34.1%)	3 (9.7%)	16 (30.2%)
***p*-value**	< 0.001		0.171 (Fisher)		0.003 (Fisher)		0.056		0.061 (Fisher)	

**Fig. 1:**
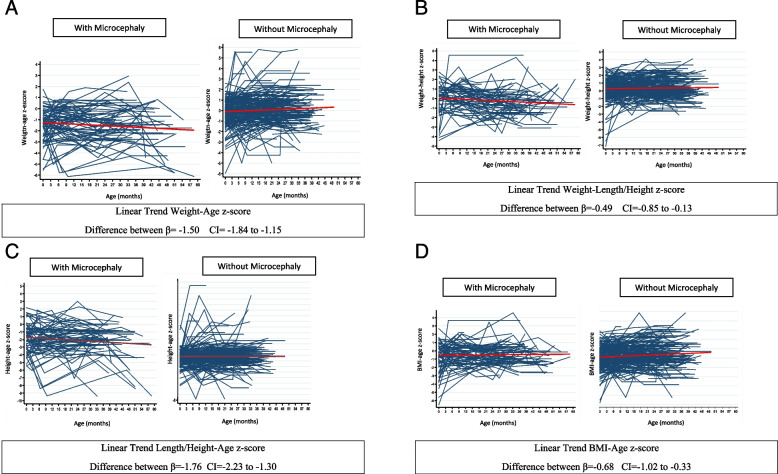
Evolution of the anthropometric indices, through linear trend analysis, of children born to women exposed to the *Zika virus, with and without microcephaly, Pernambuco/Brazil, 2016–2020. **A** Assessment of the linear trend, in the comparison between groups, of the weight-age z-score from birth to age 37–45 months, of children born to women exposed to the Zika virus, with and without microcephaly, Pernambuco- Brazil, 2016- 2020. **B** Linear trend, in the comparison between groups, of the weight-height z-score from birth to age 37–45 months, of children born to women exposed to Zika virus, with and without microcephaly, Pernambuco/Brazil, 2016–2020. **C** Linear trend, in the comparison between groups, of the height-age z-score from birth to age 37–45 months, of children born to women exposed to Zika virus, with and without microcephaly, Pernambuco/Brazil, 2016–2020. **D** Linear trend, when comparing groups, from BMI-age z-score at birth to age 37–45 months, of children born to women exposed to Zika virus, with and without microcephaly, Pernambuco/Brazil, 2016–2020

In the longitudinal analyses of weight, children with microcephaly presented with a monthly decrease in the weight-for-age z-score of -0.006 (95%CI -0.010 to 0.002; *p* = 0.050). In contrast, children without microcephaly presented with a monthly increase in the weight-for-age z-score of 0.012 (95%CI 0.005 to 0.018; *p* = 0.000). The difference between the two groups was significant: -1.50 (95%CI -1.84 to -1.15; *p* = 0.000) (Fig. 1A). When weight was considered in relation to length/height, no significant changes in the mean monthly weight-length/height z-score in children with (*p* = 0.087) or without microcephaly (*p* = 0.075). A drop in the z-score was observed up to approximately 30 months of age, followed by growth from this age onwards. The difference between the groups was -0.49 (95%CI -0.85 to -0.13; *p* = 0.007) lowest for the group with microcephaly (Fig. 1B).

In longitudinal analyses of length/height, children with microcephaly presented with a monthly decrease in the length/height-age z-score of -0.01 (95%CI -0.02 to -8.28e-06; *p* = 0.050) (Fig. 1C). In contrast, children without microcephaly presented with a monthly increase in the length/height-age z-score of 0.006 (95%CI -0.002 to 0.016; *p* = 0.143).

In longitudinal analyses of BMI, children with microcephaly presented with no significant change in BMI-age z-scores (0.01, 95%CI -0.005 to 0.02; *p* = 0.189) (Fig. 1D). In contrast, children without microcephaly presented with a monthly increase in the BMI-age z-score of 0.01(95%CI 0.005 to 0.017; *p* < 0.001). Notably, on average over the duration of follow-up, BMI-age z-scores were significantly lower among children with microcephaly compared to children without microcephaly (-0.68, 95%CI -1.02 to -0.33; *p* < 0.001).

## Discussion

In this study, children with ZIKV-related microcephaly differed from children without microcephaly in terms of weight and prematurity and presented deficits in the growth process during the study period. The frequency of birth weight of less than 2500 g, of weight-for-age z-score indicating low weight, of length/height-for-age z-score indicating very short stature, and of weight-for-length/height z-score indicating thinness was higher in children with Zika-related microcephaly. The growth curves were different between the groups with and without microcephaly, with significant deficits in the first group. The frequency of prematurity also differed between groups, and was higher among children with microcephaly.

The frequency of prematurity in children with microcephaly was 20.63%, while in children without microcephaly it was 8.78% (*p* = 0.001). A higher frequency of prematurity in children with microcephaly (27%) was also observed in another study conducted by our group [16]. In children without microcephaly, the frequency of prematurity was similar to the general population of Brazil (7.2%) [17].

The occurrence of low birth weight in children without microcephaly (8.99%) was similar to that described by the Brazilian Ministry of Health (8%) [17]. In children with microcephaly, this frequency was 31.43%, which could be related to intrauterine growth retardation, as suggested by Brasil et al [18].

The frequency of breastfeeding was high both in children with microcephaly (86.96%) and in children without microcephaly (92.5%), as was the duration of breastfeeding, which continued until the sixth month and beyond. The breastfeeding rate in Brazil, in August 2020, was 54% [19]. Although some studies have indicated the presence of the Zika virus in breast milk, the Brazilian Ministry of Health has recommended that breastfeeding should continue for mothers with ZIKV infection, taking into account that the studies have not indicated transmissibility via this route [20]. The protocols and guidelines for the care of these children may justify the high rate of breastfeeding in this population. In addition, the children received long-term medical follow-up, during which breastfeeding was encouraged. This justifies the fact that the breastfeeding rate of mothers who gave birth to children with microcephaly and of those who had Zika during pregnancy and had children without microcephaly was higher than the national rate [20].

With regard to the feeding route of children after being discharged from the maternity hospital, we observed that most children from both groups left the maternity hospital receiving exclusive breastfeeding. It is important to mention that the Global Strategy for Infant and Young Child Feeding, created in 2002 by the United Nations Children’s Fund (Unicef) and the World Health Organization (WHO), in addition to the creation of the strategy for the Hospital Amigo da Criança (Friends of Children Hospitals), by the Brazilian Ministry of Health, may have generated positive results for initiating and continuing with breastfeeding in general [21].

In terms of requiring an alternative feeding route on discharge from the maternity hospital, we observed that the frequency in both groups was less than 5%, i.e., despite the brain damage of children with microcephaly, their swallowing function was normal during the neonatal period.

In our study, the frequency of food allergy and intolerance was low in the assessments of both groups throughout the period. This result is consistent with studies that suggest an association between a high frequency of breastfeeding and a reduction in allergies and intolerance [22].

In later assessments, especially after 12 months of age, children with microcephaly in the study required the use of an alternative feeding route more often than children without microcephaly. We also observed that 16 to 18% of these children with microcephaly progressed from a temporary alternative route to a definitive alternative route (GTT). The use of an alternative feeding route may be directly linked to the need for adequate nutritional support and/or difficulties in food intake. The NET is indicated for short periods of up to six weeks and after this period, if there is no improvement, a route which is considered definitive should be chosen, which is gastrostomy [22].

The use of an alternative feeding route is directly related to the presence of dysphagia. Dysphagia occurs at a frequency that varies between 48 and 88.9% in children with microcephaly, resulting from neurological alterations that cause swallowing difficulties [15, 23, 24]. Swallowing in the first weeks of life, even in children with severe brain damage, occurs reflexively, which is why the onset of dysphagia occurs later, when the swallowing process would be governed by the cerebral cortex [25].

In our study, we observed that in the second assessment, when children were aged between 1 and 12 months, 50% of those with microcephaly presented dysphagia, as opposed to 8.7% of those without microcephaly. In the following periods, the frequency of dysphagia increased in children with microcephaly (59.4%, 62.2% and 66.7%) while in the group without microcephaly there was a reduction. We found no studies that describe the findings of dysphagia over time, although other studies have suggested that the presence of dysphagia in children with microcephaly has a frequency greater than 50% [23–25].

The results on the growth trajectory of the children assessed in this study demonstrated that children with microcephaly, when compared to children without microcephaly, in the assessment through the z-score in successive cross-sections, presented a higher frequency of very low weight and low weight in weight-for-age, of being very short and short in length/height-for-age (*p* < 0.000), with the exception of the period from 13 to 24 months. In the assessment of weight-for-length/height and BMI-for-age, there was no difference between the groups. In turn, Soares et al. monitoring children born to women both exposed and not exposed to ZIKV infection during pregnancy until 3 months of age, described that weight and length, arm circumference, arm muscle circumference and fat-free mass were different between children in the two groups in the third month of life and suggested changes in the nutritional status of children born to women exposed to the Zika virus [26]. Another study, in children with microcephaly aged between 12 and 23 months, observed changes in the z-score from length to age [27]. Despite some differences in the variables and groups compared in these studies, the results seem to converge to a nutritional deficit in children exposed to congenital ZIKV infection.

We observed differences in the monthly growth for weight-for-age, weight-for-length/height and length/height-for-age, observing that the group of children with microcephaly demonstrated a decrease in the z-score during the follow-up period. For the parameters weight-for-age and length-for-age, the z-score remained constant until 20–40 months, starting to decrease in the group of children with microcephaly from then on. For the weight-for-height parameter, the drop in the z-score started to occur after 30 months of age. The inadequacy of the abovementioned parameters may be related to insufficient caloric intake. Appropriate nutrition involves exclusive breastfeeding until the sixth month, with the introduction of solid food from that point onwards and a family menu for children from 24 months onwards [27]. Taking into account the presence of dysphagia in children with microcephaly, it may be stated that the difficulty in eating some foods may justify the nutritional deficit of these children when compared to those without microcephaly, especially after the breastfeeding period. Another possible explanation for some cases is endocrinological disorders associated with brain damage that affects the hypothalamic-pituitary axis, which may also cause then to be short [28].

The growth curves recommended by the WHO [11] are widely used tools for monitoring child growth, since they enable an accurate assessment of growth trajectories and nutritional monitoring. They serve as a basis for formulating health policies for specific groups of children. Finding an index that most closely approximates children with specific characteristics to a pattern of normality is crucial for clinical practice. In this study, we observed that there was no difference in the BMI-age index between the groups of children with and without microcephaly, and therefore this index may become a tool to assess children with microcephaly. However, we would admit that one limitation of the research is the fact that we did not assess the body composition, and therefore, this indicator may be valorizing a greater fat mass.

In a study carried out in the Northeast region of Brazil with children presenting congenital infection with and without microcephaly, Cavalcante et al. observed that the mean weight-for-age, weight-for-length/height and length/length-for-age z-scores of children with microcephaly tended to decrease slightly after birth up to 36 months. Moreover, they reported that the mean length-for-age z-cores differed significantly between the two groups at six, 12, and 24 months of age, and consistent decreases in z scores were observed in children with and without microcephaly. In the present study, it was also observed that children with microcephaly presented with malnutrition until 12 months of age, after which they remained stable [28]. In our study [29], we observed weight stability up to approximately 30 months, after which there was a drop. This result indicates that there are losses in the nutritional status of children with microcephaly in both studies.

In another study, only involving children with microcephaly, the weight-for-age z-score was -1.12 at birth and decreased by -0.08 per month, while the length-for-age z-score was -1.57 at birth, and decreased by –0.16 per month [29]. This finding brings a similar result to our study that detected a loss in the parameters of the z-score of weight and height of children with microcephaly of -0.001, although it was not statistically significant.

Although there are specific charts for children with neurological impairment, taking into account that the ZIKV infection and the children affected by it are still being characterized, we prefer to adopt the assessment suggested by the WHO and the Brazilian Ministry of Health to monitor the growth pattern of children from 0 to 5 years. Furthermore, WHO accepts the use of a common international framework, particularly in countries that do not have their own chronologically and methodologically updated framework, with the aim of reducing costs and enabling comparisons between different population groups [30, 31].

This investigation has presented strengths and limitations. The study compared two groups with intrauterine exposure to ZIKV, but with different clinical conditions, expanding the view of the growth trajectory of these children. The broad inclusion criteria allowed us to assess growth with the criteria adopted by WHO and the Brazilian Ministry of Health, across the entire spectrum of congenital Zika syndrome. One of the main limitations of this study is the lack of other measures that could provide data on body composition, as well as the lack of an assessment on food/calories intake by the study population, which could be compared to the nutritional needs of children in both groups. Thus, we suggest studies that may assess children regarding these aspects, in both groups. The description of the z-score behavior over time in addition to the β calculation enabled a greater understanding of how these parameters developed over time. Another limitation is that our investigation does not have a control group representative of the general population. It occurred because it started just after the microcephaly epidemic was identified, and children with microcephaly or born to ZIKV exposed mothers were prioritized to be followed. However, comparing children with microcephaly with children born to ZIKV exposed mothers without microcephaly, we were able to show important differences in the group with Zika-related microcephaly. Another concern is the loss of follow-up during the study period, due the long time of the study. To deal with this problem we used two different approaches: repeated cross-sectional analyses and multilevel mixed effects linear regressions with child-specific random effects.

We have concluded that children with Zika-related microcephaly remained with nutritional parameters, i.e., weight-for-age, weight-for-length/height and length/height-for-age below those presented by children without microcephaly. Following both groups over time will allow us to understand the evolution of dietary characteristics and growth parameters and provide support for defining therapeutic and care strategies.

## Data Availability

In accordance with the standards for good clinical research of the HUOC/Procape Hospital Complex Ethics Committee, data cannot be shared publicly because the dataset contains sensitive human subject data. Participants did not provide consent for public sharing of their data, and public availability would compromise patient privacy. De-identified data can made available upon reasonable request from qualified investigators by contacting email address ppg.cienciasdasaude@upe.br.

## Abbreviations

CP: Cephalic perimeter
CZS: Congenital Zika Syndrom
CDC: Centers for Disease Control and Prevetion
MERG-PC: Microcephaly Epidemic Research Group Pediatric Cohort
ZIKV: Zika virus
WHO: World Health Organization
WLZ: Weight-length/height
WAZ: Weight-age
LAZ: Length/height-age
BMI/A: Body mass index-age
HC: Head circumference
qRT-PCR: Quantitative reverse transcription polymerase chain reaction
PRNT_50_: Plaque reduction neutralization
FAV: Fundação Altino Ventura
HUOC: Hospital Universitário Oswaldo Cruz
NGT: Nasogastric tube
NET: Nasoenteral tube
GTT: Gastrostomy
PAD-PED: Dysphagia assessment protocol- pediatric

## References

[1] Eickmann SH, Carvalho MD, Ramos RC, Rocha MA, Linden VV, Silva PF (2016). Síndrome da infecção congênita pelo vírus Zika [Zika virus congenital syndrome]. Cad Saúde Pública.

[2] Medeiros AMC, Jardim-Botelho A, Santos EMS, Lopes ASA, Santos FB, Sá TPL (2021). Métodos de alimentação e evolução do peso de recém-nascidos com microcefalia congênita por Zika Vírus. Audiol Commun Res.

[3] Sobral da Silva PF, Eickmann SH, Ximenes RADA, Martelli CMT, Brickley EB, Lima MC (2021). Neurodevelopment in children exposed to Zika virus: What are the consequences for children who do not present with microcephaly at birth?. Viruses.

[4] Sobral da Silva PF, Eickmann SH, Ximenes RADA, Montarroyos UR, Lima M, Martelli CMT (2020). Pediatric neurodevelopment by prenatal Zika virus exposure: a cross-sectional study of the microcephaly epidemic research group cohort. BMC Pediatr.

[5] Araújo LA, Silva LR (2013). Anthropometric assessment of patients with cerebral palsy: which curves are more appropriate?. J Pediatr (Rio J).

[6] Moura da Silva AA, Ganz JS, Sousa PD, Doriqui MJ, Ribeiro MR, Branco MD (2016). Early growth and neurologic outcomes of infants with probable congenital Zika virus syndrome. Emerg Infect Dis.

[7] França TLB, Medeiros WR, Souza NL, Longo E, Pereira SA, França TBO, Sousa KG (2018). Growth and development of children with microcephaly associated with congenital Zika virus syndrome in Brazil. Int J Environ Res Public Health.

[8] Miranda-Filho DB, Brickley EB, Ramond A, Martelli CMT, Sanchez CN, de Araújo TBV (2021). The microcephaly epidemic research group paediatric cohort (MERG-PC): a cohort profile. Viruses.

[9] França GV, Schuler-Faccini L, Oliveira WK, Henriques CM, Carmo EH, Pedi VD, Nunes ML (2016). Congenital Zika virus syndrome in Brazil: a case series of the first 1501 livebirths with complete investigation. Lancet.

[10] Villar J, Cheikh LI, Victora CG, Ohuma EO, Bertino E, Altman DG (2014). International fetal and newborn growth consortium for the 21st century (INTERGROWTH-21st). International standards for newborn weight, length, and head circumference by gestational age and sex: the newborn cross-sectional study of the INTERGROWTH-21st project. Lancet.

[11] WHO (2006). Department of Nutrition for Health and Development. WHO child growth standards: length/height-for-age, weight-for-age, weight-for-length, weight- for height and body mass index-for-age: methods and develop.

[12] Ximenes RAA, Miranda-Filho DB, Brickley EB, Montarroyos UR, Martelli CMT, Araújo TVB (2019). Microcephaly Epidemic Research Group (MERG). Zika virus infection in pregnancy: Establishing a case definition for clinical research on pregnant women with rash in an active transmission setting. PLoS Negl Trop Dis.

[13] WHO. Anthro for personal computers, version 3.2.2, 2011: Software for assessing growth and development of the world’s children. Geneva: WHO; 2010. Disponível em: http://www.who.int/childgrowth/software/en/.

[14] Almeida FCF, Bühler KEB, Limongi SCO (2014). Protocolo de avaliação clínica da disfagia pediátrica (PAD-PED) [Portuguese].

[15] Oliveira DMDS, Miranda-Filho DB, Ximenes RAA, Montarroyos UR, Martelli CMT, Brickley EB (2021). Comparison of oropharyngeal dysphagia in Brazilian children with prenatal exposure to Zika virus with and without microcephaly. Dysphagia.

[16] Araújo TVB, Ximenes RAA, Miranda-Filho DB, Souza WV, Montarroyos UR, de Melo APL (2018). Association between microcephaly, Zika virus infection, and other risk factors in Brazil: final report of a case-control study. Lancet Infect Dis.

[17] Brasil. Ministério da Saúde. Secretaria de Atenção à Saúde. Departamento de Ações Programáticas Estratégicas. Atenção à saúde do recém-nascido: guia para os profissionais de saúde / Ministério da Saúde, Secretaria de Atenção à Saúde, Departamento de Ações Programáticas Estratégicas. 2.ed. atual. Brasília: Ministério da Saúde, 2014.

[18] Brasil P, Pereira Junior JP, Moreira ME, Nogueira RMR, Damasceno L, Wakimoto M (2016). Zika virus infection in pregnant women in Rio de Janeiro. N Engl J Med.

[19] UFRJ (2020). Universidade Federal do Rio de Janeiro. Estudo Nacional de Alimentação e nutrição Infantil – ENANI-2019: Resultados preliminares – Indicadores de aleitamento materno no Brasil.

[20] Sociedade Brasileira de Pediatria. Departamento científico de aleitamento materno. Guia Prático de Atualização. Doenças maternas infecciosas e amamentação. [Internet]. 2017 []. Available at: https://www.sbp.com.br/fileadmin/user_upload/Aleitamento_-DoencMat_Infec_e_Amam.pdf. Accessed 27 Jul 2020.

[21] Fundo das Nações Unidas para a Infância (2008). Iniciativa Hospital Amigo da Criança: revista, atualizada e ampliada para o cuidado integrado: módulo 1: Histórico e implementação / Fundo das Nações Unidas para a Infância.

[22] Minard G (1994). Enteral access. Nutri Clin Pract.

[23] Faria JBC (2020). Achados miofuncionais orofaciais em crianças com microcefalia. Distúrb Comun.

[24] Leal MC, van der Linden V, Bezerra TP, de Valois L, Borges ACG, Antunes MMC (2017). Characteristics of dysphagia in infants with microcephaly caused by congenital Zika virus infection, Brazil, 2015. Emerg Infect Dis.

[25] de Paula GL, da Silva GA, e Silva EJ, Lins MD, de Sousa Martins OS, da Silva Oliveira DM, de Santana Ferreira E, de Castro Antunes MM (2022). Vomiting and Gastric Motility in Early Brain Damaged Children With Congenital Zika Syndrome. J Pediatr Gastroenterol Nutr.

[26] Soares F, Abranches AD, Villela L, Lara S, Araújo D, Nehab S (2019). Zika virus infection in pregnancy and infant growth, body composition in the first three months of life: a cohort study. Sci Rep.

[27] Dos Santos SFM, Soares FVM, de Abranches AD, da Costa ACC, Moreira MEL, Fonseca VM (2019). Infants with microcephaly due to ZIKA virus exposure: nutritional status and food practices. Nutr J.

[28] Cavalcante TB, Ribeiro MRC, Sousa PDS, Costa EPF, Alves MTSSBE, Simões VMF (2021). Congenital Zika syndrome: Growth, clinical, and motor development outcomes up to 36 months of age and differences according to microcephaly at birth. Int J Infect Dis.

[29] Moura da Silva AA, Ganz JS, Sousa PD, Doriqui MJ, Ribeiro MR, Branco MD (2016). Early growth and neurologic outcomes of infants with probable congenital Zika virus syndrome. Emerg Infect Dis.

[30] Brasil. Ministério da Saúde. Secretaria de Atenção à Saúde. Departamento de Atenção Básica. Orientações para a coleta e análise de dados antropométricos em serviços de saúde: Norma Técnica do Sistema de Vigilância Alimentar e Nutricional - SISVAN / Ministério da Saúde, Secretaria de Atenção à Saúde, Departamento de Atenção Básica. Brasília: Ministério da Saúde; 2011.

[31] Sociedade Brasileira de Pediatria (2021). Manual de avaliação nutricional 2ª edição – atualizada - 2021/ sociedade Brasileira de Pediatria. Departamento Científico de Nutrologia.

